# Gene Editing-Based Technologies for *Beta-hemoglobinopathies* Treatment

**DOI:** 10.3390/biology11060862

**Published:** 2022-06-04

**Authors:** Ilnaz Rahimmanesh, Maryam Boshtam, Shirin Kouhpayeh, Hossein Khanahmad, Arezou Dabiri, Shahrzad Ahangarzadeh, Yasaman Esmaeili, Elham Bidram, Golnaz Vaseghi, Shaghayegh Haghjooy Javanmard, Laleh Shariati, Ali Zarrabi, Rajender S. Varma

**Affiliations:** 1 Applied Physiology Research Center, Cardiovascular Research Institute, Isfahan University of Medical Sciences, Isfahan 73461-81746, Iran; ilnazrahimmanesh@gmail.com (I.R.); arezou_dabiri@yahoo.com (A.D.); shaghayegh.haghjoo@gmail.com (S.H.J.); 2 Isfahan Cardiovascular Research Center, Cardiovascular Research Institute, Isfahan University of Medical Sciences, Isfahan 81583-88994, Iran; maryamboshtam@gmail.com (M.B.); golnazvaseghi@yahoo.com (G.V.); 3 Erythron Genetics and Pathobiology Laboratory, Department of Immunology, Isfahan 76351-81647, Iran; shirin_ake@yahoo.com; 4 Department of Genetics and Molecular Biology, School of Medicine, Isfahan University of Medical Sciences, Isfahan 73461-81746, Iran; hossein_khanahmad@yahoo.com; 5 Infectious Diseases and Tropical Medicine Research Center, Isfahan University of Medical Sciences, Isfahan 73461-81746, Iran; shahrzadahangar@yahoo.com; 6 Biosensor Research Center, School of Advanced Technologies in Medicine, Isfahan University of Medical Sciences, Isfahan 73461-81746, Iran; esmaeili161@gmail.com (Y.E.); elhambidram@gmail.com (E.B.); 7 Department of Biomaterials, Nanotechnology and Tissue Engineering, School of Advanced Technologies in Medicine, Isfahan University of Medical Sciences, Isfahan 73461-81746, Iran; 8 Cancer Prevention Research, Isfahan University of Medical Sciences, Isfahan 73461-81746, Iran; 9 Department of Biomedical Engineering, Faculty of Engineering and Natural Sciences, Istinye University, Sariyer, Istanbul 34396, Turkey; alizarrabi@gmail.com; 10 Regional Centre of Advanced Technologies and Materials, Department of Physical Chemistry, Faculty of Science, Palacky University, Šlechtitelů 27, 783 71 Olomouc, Czech Republic

**Keywords:** beta-thalassemia, gene therapy, ZFN, TALEN, CRISPR

## Abstract

**Simple Summary:**

β-thalassemia syndromes are clinically and genetically heterogeneous blood disorders presented by β-chain deficiency in hemoglobin production. Despite improvements in transfusion practices and chelation treatment, many lingering challenges have encouraged researchers to develop newer therapeutic strategies such as gene editing. One of the most powerful arms of genetic manipulation is gene editing tools, which have been recently applied to improve β-thalassemia symptoms. Nevertheless, several obstacles, such as off-target effects, protospacer-adjacent motif requirement, efficient gene transfer and expression methods, DNA-damage toxicity, and immunotoxicity issues still need to be addressed in order to improve the safety and efficacy of the gene editing approaches. Hence, additional efforts are needed to address these problems, evaluate the safety of genome editing tools at the clinical level and follow the outcomes of gene editing tools-mediated therapeutic approaches in related patients.

**Abstract:**

Beta (β)-thalassemia is a group of human inherited abnormalities caused by various molecular defects, which involves a decrease or cessation in the balanced synthesis of the β-globin chains in hemoglobin structure. Traditional treatment for β-thalassemia major is allogeneic bone marrow transplantation (BMT) from a completely matched donor. The limited number of human leukocyte antigen (HLA)-matched donors, long-term use of immunosuppressive regimen and higher risk of immunological complications have limited the application of this therapeutic approach. Furthermore, despite improvements in transfusion practices and chelation treatment, many lingering challenges have encouraged researchers to develop newer therapeutic strategies such as nanomedicine and gene editing. One of the most powerful arms of genetic manipulation is gene editing tools, including transcription activator-like effector nucleases, zinc-finger nucleases, and clustered regularly interspaced short palindromic repeat–Cas-associated nucleases. These tools have concentrated on γ- or β-globin addition, regulating the transcription factors involved in expression of endogenous γ-globin such as KLF1, silencing of *γ-globin* inhibitors including BCL11A, SOX6, and LRF/ZBTB7A, and gene repair strategies. In this review article, we present a systematic overview of the appliances of gene editing tools for β-thalassemia treatment and paving the way for patients’ therapy.

## 1. Introduction

β-thalassemia syndromes are clinically and genetically heterogeneous blood disorders presented by β-chain deficiency in hemoglobin production [1]. Phenotypes of β-thalassemia are highly varied, from an asymptomatic disorder to severe anemia, β-thalassemia major or Cooley’s anemia, which causes death before the age of 10 and the only therapeutic approach available is regular transfusion of red blood cells (RBCs) [2,3].The annual incidence rate of symptomatic β-thalassemia is estimated to be 1 per 100,000 live births in the world. The origins of β-thalassemia were found to be in the Mediterranean, while its major types are mostly seen in the Middle East, Southeast Asia, India, and China. Moreover, increased prevalence of β-thalassemia has been reported in malaria endemic countries. Notably, human migration has contributed to the further spread and establishment of β-thalassemia all around the world [4].

**Figure 1:**
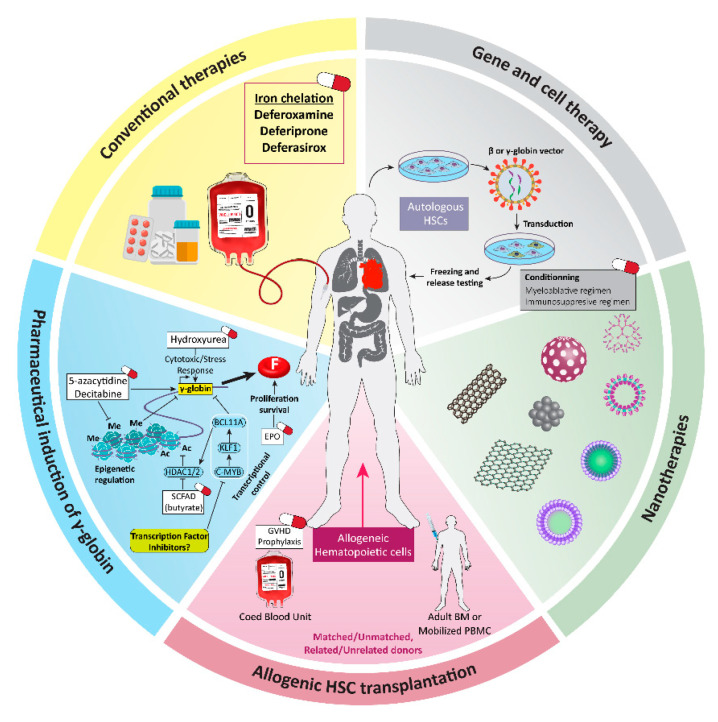
Current and future therapeutic approaches for β-thalassemia major. Among the various kinds of treatments used for treatment of β-thalassemia patients, nanomedicine and gene therapy are the new emerging ones and have provided new hope in treatment of patients. Their application does not require immunosuppression.

Allogeneic bone marrow transplantation (BMT) from a matched donor is the traditional treatment for β-thalassemia major. However, significant disadvantages of BMT, including the limited HLA-matched donors, the need for a long-term immunosuppressive regimen, the limited application of BMT in young patients, and further immunological side effects, have limited its use [5,6] (Figure 1).

The β-thalassemia major management procedure is a combination of lifelong RBC transfusion and chelation therapy to restrict iron deposition, and full observance of this treatment greatly increases the life expectancy of the patients [7]. This therapeutic approach is too expensive in many countries; therefore, novel therapeutics should be introduced in treatment of this disease. The majority of patients with β-thalassemia major die from heart failure due to secondary hemochromatosis following sub-optimal iron chelation, or hepatocarcinoma in older patients [8] (Figure 1).

Other strategies for β-thalassemia major treatment are epigenetic approaches that intervene in the fetal gamma globin reactivation. Increased synthesis of hemoglobin F (HbF) is accompanied by significant decrease in intensity of thalassemia major’s symptoms [9]. There are several chemical compounds inducing gamma globin reactivation including 5-azacitidine as a demethylating agent and derivatives of small-chain fatty acids, such as arginine butyrate, which are more effective when administered with erythropoietin (EPO) [9] (Figure 1). The conventional therapies for β-thalassemia major suffer from a number of adverse impacts, such as neurological complications and dramatically increased levels of platelets, thus encouraging researchers to develop new strategies for β-thalassemia major treatment including nanomedicine and gene editing [10].

Nanomedicine as an appropriate and new treatment with high efficacy for treatment and diagnosis of disease [10,11,12] can be used in the management of various disorders, particularly blood disease disorders (BDDs) [13]. It switches the conventional drugs/treatments into nano-platforms carrying small therapeutic molecules as newer strategies in disease treatment [14,15]. The application of nanomedicine for curing BDDs was first approved by FDA in 2018. Considering mRNA-based approaches as the first approved techniques for therapy to treat BBDs, and the risk of degradation and cellular uptake of unprotected mRNA, nanomedicine is able to protect and control mRNA function for in vivo application [16]. Direct whole blood transfusion is still the ideal treatment method for the rare BDDs [15], although, short half-life and the possibility of bacterial infection, are referred as the limitations of whole blood storage. Consequently, another possible application of nanomedicines could be synthesis/designing of artificial blood components [15]. For instance, in view of the main role of RBCs as a gas (oxygen and carbon dioxide) transporter, RBC alternatives might be considered as a solution for urgent situations in BDDs and particularly thalassemia treatment. Nanomedicine can imitate the major features of the RBCs by incorporating the functional parts of the molecule into the nanoplatforms [15]. Despite the advantages of nanomedicines, the possible toxicity and complications in size determination, as well as the generalization of the functional modules, also need to be considered [17] (Figure 1).

Gene therapy is an effective one-time treatment method, which does not require immunosuppression and graft versus host disease (GVHD) prophylaxis and can be employed for every patient. Gene therapy is mainly applied for β-thalassemia treatment to attain stable functional globin genes or manipulation of transcription factors regulating gamma chain-expression in the patient’s own hematopoietic stem cells (HSCs) to modify inefficient erythropoiesis and to treat hemolytic anemia [18]. One of the most powerful arms of genetic manipulation is gene editing tools (GETs), which have been recently applied to improve β-thalassemia symptoms. Herein, we deliberate a systematic overview on the applications of GETs for β-thalassemia treatment in recent years.

## 2. β-Thalassemia: Molecular Basics

β-globin is typically codified by a group of β-like globin genes including ε (*HBE*), Gγ (*HBG2*), Aγ (*HBG1*), δ (*HBD*), and β (*HBB*). These genes are located on the chromosome 11(11p 15.15) in order to make various tetramers of Hb such as embryonic Hbs (Hb Gower-1 (ζ_2_ε_2_), Hb Gower-2 (α_2_ε_2_), and Hb Portland (ζ_2_β_2_)), fetal Hb (α_2_γ_2_), and adult Hbs (HbA, α_2_β_2_ and HbA_2,_ α_2_δ_2_) [7]. Hemoglobin genes are expressed at distinct growth phases via a hemoglobin switching from embryonic to fetal and finally to adult. Moreover, the globin gene expression including fetal genes relies on vital regulatory regions within the globin domain, including local promoter sequences and the control region of *β-globin* locus located at the upstream β-globin containing sites that are hypersensitive to DNase 1. Indeed, some particular erythroid transcription factors including GATA-binding factor 1 (GATA-1) and -2, Kruppel Like Factor 1 (KLF1), Nuclear Factor, Erythroid 2 (NF-E2), Erythroid 2 (NF-E4), Stem Cell Leukemia (SCL), BAF Chromatin Remodeling Complex Subunit *BCL11A* (*BCL11A*) and different cofactors such as p300 and Friend of GATA (FOG) bind to the regulatory regions of the globin gene [19]. Furthermore, a number of proteins, including the DRED complex, IKAROS, and GATA-1, have been demonstrated to bind to the -*globin* promoter region and suppress transcription of fetal genes. BAF Chromatin Remodeling Complex Subunit *BCL11A* (*BCL11A*) and SRY (sex determining region Y)-box 6 (SOX6) are also involved in the fetal and embryonic globin genes being silenced. These two proteins are most likely part of a bigger protein complex that also includes the NuRD and GATA-1 corepressor complexes.

There are various molecular mechanisms underlying the β-globin downregulation. Typically, mutations could arise at the early fetal stage, leading to the complete deletion of a globin gene and causing β^0^-thalassemia. Based on the level of deletion in the β-chain, various mutations produce β-globin subunits with different reduced expression levels which are classified as β^+^ or β^++^ (“silent”) thalassemia. In fact, the reduced β-globin chain production causes excess unassembled α-globin chains in erythrocyte precursors to aggregate, and further drives the pathophysiology of the disorder [20]. Thus, the severity of the disorder is typically dependent on the balance of α- and β-globin chain production as well as the amount of the free α-chain. Moreover, structurally abnormal β-chain variants following point mutations in the β-globin β-globin gene are extremely unstable and cause a type of β-thalassemia (HbE [β26 Glu→Lys]), known as “thalassemic hemoglobinopathies” [21]. Likewise, some variants of β-globin chain are not capable of generating a stable form of hemoglobin tetramers, resulting in a defect in β-globin function. Notably, these β alleles are rare, with a dominant inheritance pattern leading to severe anemia. In contrast, common types of β-thalassemia are inherited as haploinsufficient Mendelian recessives, where two copies of β-thalassemia alleles are essential for developing the disorder [22].

**Figure 2:**
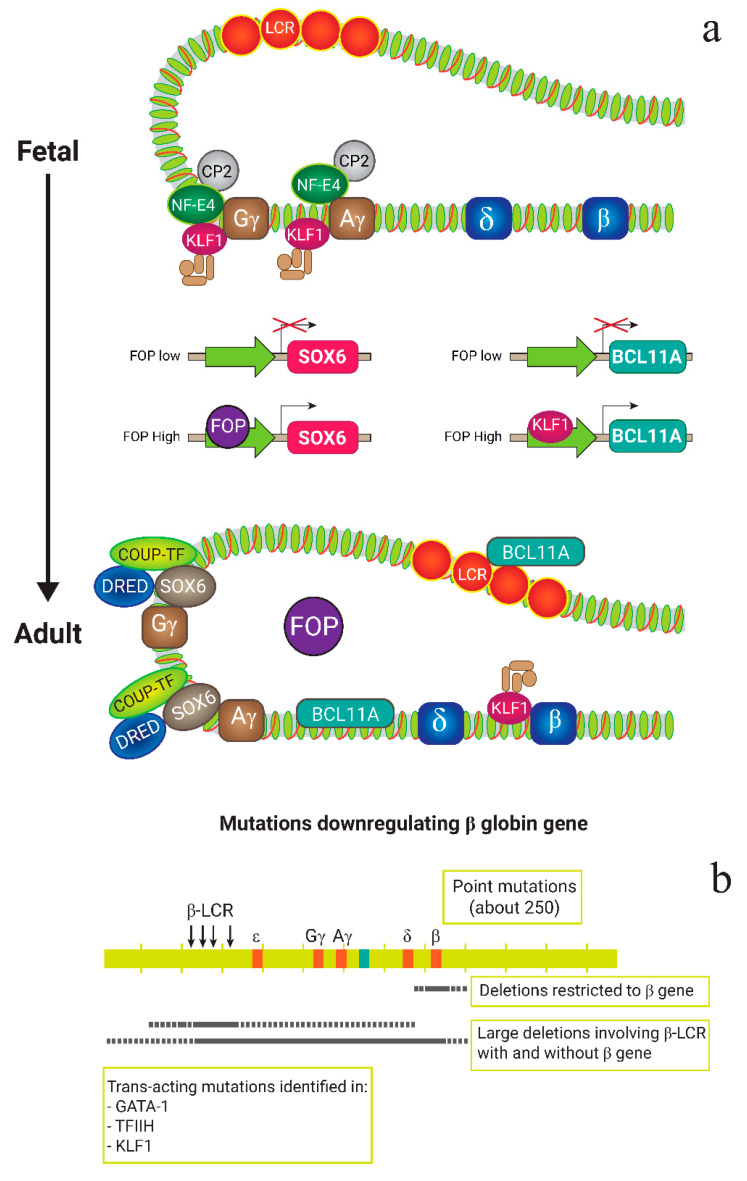
Molecular mechanisms underlying β-thalassemia. (**a**) Role of fetal globin repressors including BCL11A, SOX6 and KLF1 in the expression of γ and β-globin gene: In the fetus, chromatin factor Friend of Prmt1 (FOP) expression is low. Hence, fetal globin repressors including BCL11A, SOX6 and KLF1 did not have any function, and transcription factors such as NF-E4 bind the coding region of the gene and fetal globin (HbF) is synthesized. In adults, expression of FOP is high and fetal globin repressors are activated, bind to the coding site of the gene and β-globin is produced in erythroid progenitors. (**b**) In β-thalassemia, some mutations cause β-globin gene to downregulate, which are known as cis and trans acting elements, leading to downregulation of β gene expression. Mutations in GATA-1, TFIIH, and KLF1 are known as trans acting regulatory elements, while mutations in alleles of *β-globin* locus are known as cis acting elements.

Most of the β-globin downregulating-related mutations occur in the *β-globin* locus alleles, which are known as *cis*-acting regulatory elements, while mutation in *trans*-acting elements modifies the β-globin gene expression and leads to a phenotype that segregates independent of the β-globin cluster. Nearly 300 alleles of β-thalassemia have been identified to date [23]. Although the majority of the phenotypes in α-thalassemia are related to deleterious mutations in the α-globin gene cluster, the majority of β-thalassemia phenotypes appear by mutations in one or more nucleotides in the β-globin cluster or instant flanks [24]. Figure 2 illustrates the summary of molecular mechanisms underlying β-thalassemia.

## 3. Gene Editing Tools

**Figure 3:**
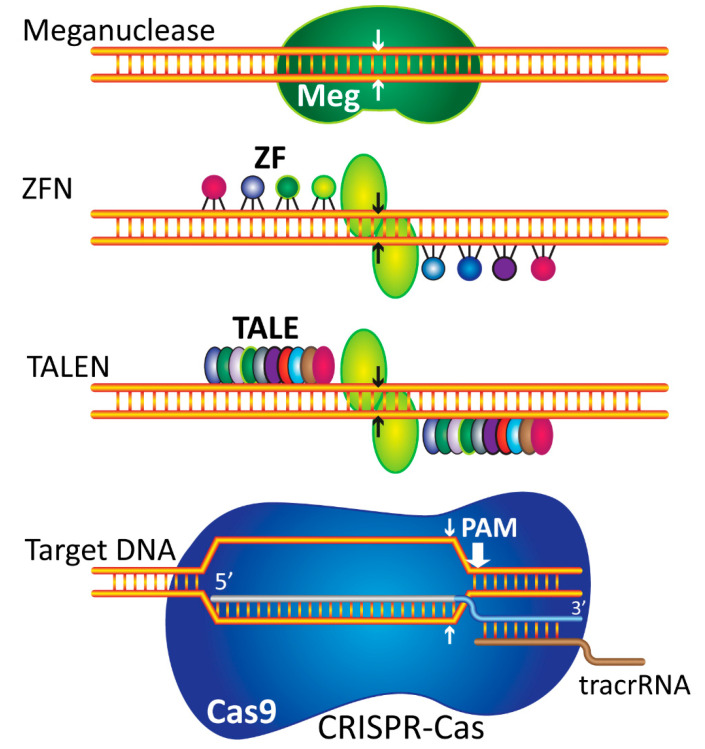
Gene editing tools. This figure reveals the mechanisms of targeted nucleases. From top to bottom: Meganucleases or homing endonucleases are nuclease enzymes that do not have separate DNA binding and cleavage domains, and recognize a 20–40 bp DNA sequence. Meganucleases may be utilized in all genome types to repair damaged genes in gene therapy by interrupting their DNA substrates as dimers.

Engineered bacterial nucleases and the creation of the programmable nucleases, has made possible editing of genome sequences. These tools include zinc-finger nucleases (ZFNs), transcription activator-like effector nucleases (TALENs) or clustered regularly interspaced short palindromic repeat (CRISPR)–Cas-associated nucleases (Figure 3) [25].

### 3.1. Zinc Finger Nucleases

ZFNs are a group of engineered and chimeric nucleases which were developed by combining a bacterial endonuclease of FokI (a double-stranded DNA nickase,) with DNA-binding zinc finger domains to target and cut a particular 3–4 bp DNA sequence site of genome [26,27]. The ZFNs are used to modify targeted sequence through creation of a double stranded nick on target DNA and induction of an indel mutation for improving the gene function. The ZFNs do not function specifically for the target sequence, and the same genomic sequences off-targets can be affected and modified by the DNA molecule of donor leading to undesired genome modification. However, these systems are capable of elevating the specificity of DNA targeting and attenuating off-target effects using dimerized FokI. [28].

### 3.2. TALENs

TALENs were initially introduced in 2011 based on the application of transcription activator-like effector (TALE) proteins found in nature (Xanthomonas species). These GETs are artificial proteins including a nonspecific nuclease (FokI) cleavage domain connected to a DNA-recognition TALE region. TALEN activity is associated with two DNA binding sites flanking an undedicated 12–20 bp spacer sequence. Based on spacer length and TALE construction, the DNA cleavage efficiency in TALENs is different.

As with ZNFs, TALENs are chimeric nucleases with a DNA binding and an effector domain. Both the ZFN and TALEN nucleases act as dimers, so a pair of ZFN or TALEN should be developed that is capable of targeting a genomic site [29]. Unlike ZFNs, which are only commercially available, engineered TALENs can be easily synthesized in any molecular biology laboratory; however, cloning of TALEN constructs can be laborious and time-consuming. The main advantages of TALENs over ZFNs are their enhanced specificity and predictability of binding to a particular sequence and cost effectiveness of TALENs. Both ZFNs and TALENs constructions require in vitro verification to demonstrate an adequate level of cleavage efficiency before they can be employed in experiments. Since each gene editing with ZFN or TALEN requires the synthesis of a new engineered nuclease, both techniques are relatively expensive and complicated [30].

### 3.3. CRISPR

CRISPR is a new GET strategy that was initially introduced with considerable ability for targeted genome engineering [31,32]. CRISPR-associated protein (Cas) is a genetic engineering technique for the correction of genomes of living organisms. This system includes three components, an endonuclease (Cas9), a sequence-specific CRISPR RNA (crRNA), and a trans-activating crRNA (tracrRNA), that links Cas9 with crRNA [33]. The CRISPR-Cas9 system has been simplified by fusion of two RNA sequences into a single 100-nucleotide chimeric RNA called gRNA or sgRNA, which can competently lead Cas9 to recognize and cut foreign DNA strands in a special target site, and a specific array of tandem reiterative elements which is separated with short variable 20 nucleotide sequences. CRISPR technology has advantages over previous systems. The most important advantage is the shorter assembly time and simplicity due to application of artificial sequences which are much easier than protein engineering required in the ZFN and TALEN systems [34]. The major problem concerning CRISPR/Cas9 application is the off-target effects. The specificity of CRISPR/Cas9 system in mammalian cells can be increased with adding two “nickase” CRISPR/Cas9 complexes, which have a specific binding region. Nickase Cas9 is created by mutation in one of the two Cas9 nuclease domains and can cleave only one of the DNA strands. The adjacent DNA site could be targeted by a pair of nCas9s to create a double-strand break (DSB). Both of these nCas9s have been applied to improve the Cas9-based genomic editing specificity [35].

## 4. HbF or Gamma Globin Induction Using Gene Editing Tools

Based on preclinical studies, HbF re-induction can improve the sickle cell disease (SCD) and β-thalassemia severity. The initial clinical observations have revealed, in the case of natural mutations in the HBB gene cluster or related genes, expression of γ-globin and, subsequently, HbF would modify this deficiency [36].

Moreover, β-thalassemic infants show symptoms after attenuating HbF production. Recently, researchers have verified the effect of high HbF synthesis on the improvement of clinical symptoms of β-thalassemic patients [37,38,39]. All together, these observations lead to further investigation of HbF inducers with emphasize on the role of naturally higher levels of HbF in β-thalassemia patients [40,41].

The fact is that β-thalassemia and SCD patients suffer from a less severe disorder when HbF is increased; therefore, HbF re-expression could be considered as a beneficial therapeutic approach for these diseases. Some animal and human studies have reported the usage of GETs for HbF elevation [42,43,44]. Strategies for HbF induction in adult RBCs deploying GETs can be summarized as inducing natural mutations regarding enhanced HbF level, knocking out repressors of HbF and modifying intermediates of epigenetic to control HbF synthesis [45].

### 4.1. Targeting the HbF Repressors

**Figure 4:**
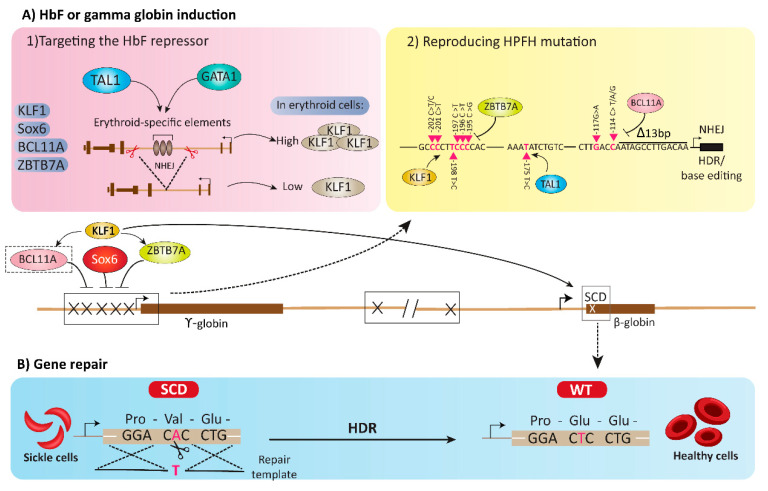
Molecular mechanism of β-globin repair in beta-hemoglobinopathies patients by genome editing tools. In adults, BCL11A, SOX6 and ZBTB7A via binding to the proximal promoter of the γ-*globin* gene cause it to repress, and KLF1 activated their expression to inhibit expression of γ-globin gene. Moreover, KLF1 as an activator, upregulates β-globin through direct binding to its promoter. (**A**) HbF upregulate by: (1) targeting erythroid–specific elements in the *KLF1*, *SOX6*, *BCL11A* and *ZBTB7A* gene, their expression is attenuated; and (2) creating a point mutation mimicking hereditary persistence of fetal hemoglobin (HPFH) phenotype via targeting the binding sites of BCL11A and ZBTB7A transcription factors in the γ-*globin* gene promoter. (**B**) The last mechanism is point mutation repair of the β-globin gene in patients with sickle cell disease or β-thalassemia and return to wild-type by homology-directed repair (HDR).

Recent advances in our knowledge of *β-globin* locus regulation at transcriptional level have revealed a strict negative control of γ-globin gene expression, which is mediated by epigenetic modification and different transcription factors [46]. Several experimental and clinical treatments have been used to increase HbF levels by direct targeting of fetal globin repressors including BAF Chromatin Remodeling Complex Subunit BCL11A (BCL11A), SRY-Box Transcription Factor 6 (SOX6), HBS1L-MYB, KLF1 and Zinc Finger and BTB Domain Containing 7A (ZBTB7A). Re-induction of HbF using GETs could possibly delete or reduce the expression of the HbF repressors; however, various types of side effects may occur since transcription factors often have broad spectrum functions to regulate multiple target genes (Figure 4A(1)).

#### 4.1.1. BCL11A

The genome wide association studies have identified BCL11A as the master repressor of HbF production in both the mouse models and human cells [47]. Erythroid-lineage Bcl11a knockout mouse SCD model showed pancellular HbF induction and phenotypic modification in the mice with minimal effects on erythropoiesis [48]. Another study revealed that microdeletions in the *BCL11A* locus of haploinsufficient patients led to considerable neurocognitive phenotypes, in addition to increased HbF level to a therapeutic extent, which modify the pathologic and hematologic deficiencies [49]. In principle, BCL11A knockout through exerting frameshift null alleles in BCL11A coding region could be a feasible treatment approach for these diseases. However, the role of BCL11A in B-cell development and HSC function are identified as the major obstacles, which has limited the application of the BCL11A knockout strategy. As BCL11A plays a critical role in lymphoid and neural development, Bcl11a deletion causes neonatal mortality [50,51]. Moreover, different supportive evidence indicated a lymphopoiesis defect in Bcl11a-knockout HSCs [52]. Based on the findings of a recent study, due to role of BCL11A coding site in the lymphoid lineage synthesis and function of hematopoietic stem cells, its genetic editing could not be considered as an efficient treating approach [53]. Thus, recent strategies have mostly focused on the deletion of BCL11A-binding sequences in the proximity of γ-globin gene or using erythroid-restricted expression of genome-editing components that have been applied for erythroid specific repression of BCL11A [54].

A recent HbF-associated GWAS demonstrated a BCL11A erythroid intronic enhancer comprising three DNase I hypersensitive sites, named +55, +58 and +62 based on the distance from the BCL11A transcription start site in kilobases [54,55]. The deletion of orthologous regions in a mouse erythroid cell line led to the deletion of BCL11A at both the RNA and protein levels, while BCL11A expression was saved in a B-cell line with the same deletion. Deletion in all intronic positions of BCL11A enhancer, but especially in the +58 position demonstrated a significant reduction in BCL11A expression and increase in γ-globin levels in human erythroid cells [54,56]. In a direct comparison between application of ZFNs to disrupt the BCL11A coding region and the erythroid-specific enhancer of BCL11A, it was revealed that the bi-allelic disruption of GATAA motif in BCL11A erythroid enhancer led to increasing levels of fetal globin expression. Simultaneously, targeting the BCL11A enhancer appears to be more tolerated within the erythroid lineage since the residual low levels of BCL11A are insufficient to repress g-globin, while promoting cellular fitness. Therefore, directed deletion of the 12-kb erythroid enhancer of *BCL11A* could function as an alternate strategy for targeting BCL11A coding sequence [57]. Aiming a 200 bp region within the human erythroid-enhancer of *BCL11A* (including GATAA motif) by CRISPR/Cas9 showed increased expression of γ-globin in the K562 cell line [58]. The investigational application of CRISPR/Cas9-based gene editing to treat a patient with β-thalassemia and the other patient with SCD has been reported in a clinical trial. In this study, the patient who received the autologous CD34+ cells edited by CRISPR/Cas9 targeting the BCL11A enhancer showed an increased level of HbF that was pancellularly distributed [59]. Recently, during a clinical trial, Franguol et al. administered CTX001 (an autologous CD34+ cells edited by CRISPR/Cas9 targeting the BCL11A enhancer) to a beta-thalassemia patient and a SCD patient and followed them up for 12 months. They succeeded to increase HBF level considerably and continuously in both patients who received CTX001 [60].

Ma et al. examined the effects of plasmid length and structure on electroporation efficiency in HSPC as the primary method used to transfect these cells. As a result, they investigated the use of a minicircle (MC-DNA) vector without a bacterial backbone to supply the CRISPR/SaCas9 tool into HSPCs to reactivate γ globin expression as a potential therapeutic approach for β thalassemia patients. They found that the transfection efficiency of CD34+ hematopoietic stem cells depends on plasmid length and linearization. Furthermore, the MC transgene expression without major plasmid sequences was excellent compared to conventional plasmids in vitro and in vivo. In this research, MC DNA was used to deliver the cassette of Staphylococcus aureus Cas9 (SaCas9) into HSPCs, and a single-guide RNA targeting the erythroid enhancer region of BCL11A was chosen. After electroporation with MC-DNA, an apparent efficiency of gene editing and reactivation of γ-globin expression was obtained in unsorted HSPC-derived erythroblasts. The developed MC-DNA vector offered a potential strategy to deliver SaCas9 cassettes and reactivate γ-globin expression to alleviate β thalassemia syndromes [61].

#### 4.1.2. SOX6

The SOX6 transcription factor plays a critical role in gene switching of β-globin in erythroid cells as well as BCL11A [62]. The first described role for SOX6 in gene regulation of β-globin was documented by the evaluation of the SOX6-knockout mice model. The expression of mice embryonic β-like globins (εy and βh1) was markedly increased at the fetal liver stage of Sox6-knockout mice model, p100H [63]. The silencing effect of BCL11A for the γ-globin gene is suggested to be through long-range cooperation with SOX6. The BCL11A-mediated suppression of γ-globin genes is exerted through both the local interactions with SOX6 chromatin-associated proteins and within the human β-globin gene. Throughout hemoglobin switching, SOX6 interacts with γ-*globulin* proximal promoters, thus mediates BCL11A recruitment to the γ-genes proximal regions [62]. In adult human erythroid lineage, γ-globin gene expression, which is mediated by stem cell factor, is also related to *SOX6* downregulation. Furthermore, CRISPR/Cas9 and ZFN technologies have recently been used to generate a mutation in the binding site of SOX6 for γ-globin gene reactivation. These studies showed that induction of mutation in the binding domain of *SOX6* gene gives rise to γ-globin reactivation [9,64]. Altogether, SOX6 appears to function as a potentially promising target for HbF reactivation.

#### 4.1.3. LRF/ZBTB7A

Another transcription factor, LRF/ZBTB7A (Pokemon), has newly been documented as a major repressor of γ-globin gene expression. LRF-knockout mice model exhibits elevated levels of Hbb-bh1 as the embryonic globin while maintaining normal levels of Hbb-y. This phenomenon is in contrast to the Bcl11a-null mice models, in which both embryonic globins, Hbb-y and Hbb-bh1, are overexpressed. Zbtb7a-/-mice models are embryonic lethal due to anemia, whereas the conditional knockout of Zbtb7a in adult mice leads to mild macrocytic anemia following inefficient terminal erythropoiesis [48,65]. CRISPR/Cas9-mediated knockout of LRF in an erythroid cell line resulted in significant upregulation of γ-globin gene expression. The LRF/BCL11A double knockout model was indicated to upregulate HbF expression up to of 90%, suggesting that the role of LRF in γ-globin regulation is moderately independent of Bcl11a. Further analysis confirmed a slight delay in erythroid lineage differentiation upon *LRF* knockdown in primary human CD34 HSPCs differentiated down to the erythroid lineage with a subsequent γ-globin induction [66]. Although the effect of *LRF* knockdown on γ-globin upregulation is prominent, the LRF role in cell fate decisions in multiple hematopoietic lineages, and specifically for terminal erythropoiesis, can limit the LRF application as a potential therapeutic strategy [67].

#### 4.1.4. KLF1

The KLF1 has long been appreciated, as a main factor in the switching of γ- to β-globin and HbF reinduction processes. Thus, some investigations have targeted KLF1 as a genetic regulator by GETs including CRISPR/Cas9 to reactivate the expression of HbF; *KLF1* gene manipulation leads to a profound effect on *KLF1* promoter sequence [68]. Our previous study was conducted to study the ability of an engineered CRISPR/Cas9 system to target *KLF1* gene to induce *KLF1* disruption and eventually stop the γ to β hemoglobin switching process in the K562 cell line.

Our results showed that the γ-globin level was significantly raised in differentiated K562 cells treated with CRISPR/Cas9 [69]. In a parallel investigation, we effectively applied the ZFN technology to the *KLF1* gene knockout through targeted genome deletion induction. We envisaged that targeted induced mutations in the *KLF1* gene coding region, lead to β-globin chain synthesis reduction and *BCL11A* gene down regulation, thus removing the inhibitory effect of BCL11A on the expression of the *γ-globin* gene [64]. Furthermore, in another comparative analysis, KLF1, BCL11A, and HBG1/2 were targeted in a parallel manner in CD34+HSPCs by CRISPR/Cas9 to induce fetal hemoglobin [70]. The results were compared to assess the impact of each targeted gene on HbF induction and safety measurements by molecular analyses to select the most effective candidate for clinical investigation. The successful downregulation of *KLF1* and *BCL11A* transcripts led to prominent *γ-globin* mRNA expression, and significant HbF levels, comparable to Hereditary Persistence of Fetal Hemoglobin (HPFH) mutations. Although the elevated level of HbF (up to 25%) after *KLF1* gene disruption was associated with no off-targets (verified by GUIDE-seq), the negative effect of *KLF1* knockdown was detected in further analysis, which could be a major safety concern in the clinical application of this strategy. When compared to non-edited reference cells, RNA-seq analysis indicated that targeting the *KLF1* gene with CRISPR/Cas9 dysregulated many genes with various biological activities [70]. Recent investigations have documented the reduced *KLF1* expression, the influence of the profile expression of genes complicated in cancer (*FLI-1*) and some biological processes, including microcytosis (*AQP1*) and cell–cell interaction (*CD44* and *ITGA2B*) [71,72].

### 4.2. Reproducing HPFH Mutations Recapitulates A Mutation Associated with A Benign Genetic Condition

Hereditary persistence of fetal hemoglobin (HPFH) is a benign condition in which some mutations naturally occur in the fetal globin promoter sequence, leading to HbF reactivation in adulthood [73]. These natural mutations consist of small deletions and/or single point mutations in the proximal promoter of fetal *γ-globin* gene [74].

A recent therapeutic strategy for β-thalassemia is to mimic HPFH mutations to reactivate HbF (Figure 4A(2)). One of the HPFH mutations is British HPFH, in which a point mutation of the −198 T > C in the fetal globin gene promoter has occurred. As HbF expression level elevates up to 20% in these thalassemic patients, the symptoms of β-hemoglobinopathies are considerably improved. In an in vitro and in vivo combinational study by Wienert and his colleagues, CRISPR/Cas9 technology was applied to exert a similar mutation to ameliorate the β-hemoglobinopathies symptoms by increasing HbF level. Subsequent to introduction of the −198 T > C mutation into the fetal globin promoter of the clonal *WT HUDEP-2* cells, the mRNA expression percent of *γ-globin* [γ/γ + β] elevated from ∼0.5–1% to 4–6%. Moreover, the number of HbF-immunostained cells improved in the −198 T > C mutated cells. Hence, deployment of CRISPR/Cas9-mediated homology-directed repair system to induce the −198 T > C mutation in clonal cells, leads to the creation of a new binding site for KLF1 as the major erythroid gene activator, which further enhances the HbF level [75].

Another type of HPFH naturally occurs through −175 T > C single point mutation, which leads to significant increase in fetal γ-globin to erythroid cell lines ratio. The elevated fetal globin level in these individuals is related to de novo recruitment of the activator TAL1 to promote chromatin looping of distal enhancers to the modified *γ-globin* promoter. In this study, the −175 T > C substitution into the *Aγ-* and *Gγ-globins* genes promoters of murine erythroid and human erythroid K562 cells was induced using TALEN-based homologous recombination strategy. In both the cell lines, TAL1 could bind to the promoters and activate γ-globin in the mutated erythroid cells [76]. Generally, the results ascertained that the percentage of the HbF was significantly increased between 16 and 41% of total hemoglobin. In a recent study on β-thalassemic Egyptians, mutation in the *β-globin* first intron (IVS-1-110 [G > A]), which is the most prevalent mutation in this country [77], was directly corrected using CRISPR/Cas9-edited non-homologous end joining (NHEJ) technology [78]. The researchers succeeded in knocking out the mutation in peripheral blood CD34+ hematopoietic stem cells of the β-thalassemic patients, and the edited cells were then differentiated to erythroid lineage in culture media in the presence of erythropoietin [78].

One of the other point mutations which is responsible for HbF re-induction and improvement of β-thalassemic patients sign and symptoms, is related to the direct *HbF* gene repressors, BCL11A and ZBTB7A. In wild-type species, BCL11A as well as ZBTB7A directly join with the *γ-globin* gene promoter at the positions –115 and –200 bp, respectively, and repress the *fetal globin* gene expression. Martyn and his colleagues employed CRISPR/Cas9 technology to exert the homozygous HPFH-associated mutations of −117 G > A, −114 C > A, ∆ 13 bp, and–195 C > G in *γ-globin* gene promoter of erythroid cells where BCL11A and ZBTB7A can bind to the gene, to prevent the repressors binding and elevate *γ-globin* gene expression; results showed increased *γ-globin* mRNA and HbF levels in –117 G > A, −114 C > A, ∆ 13 bp, and −195 C > G mutated populations [79].

## 5. Gene Repair Strategies

Gene repair, as a precise approach that potentially corrects the mutations in the native *β-globin* locus, is a novel non-pharmacological therapeutic method for the β-hemoglobinopathies treatment (Figure 4B). Over 200 various point mutations are known to cause β-thalassemia [80], while in contrast, a single point mutation of Glu > Val in the coding site (position 7) of the *β-globin* is responsible for SCD. Effective specific modifications exert on endogenous genomic loci by GETs, as recently reported for the human *globin* locus [81].

The binding of a ZFN or TALEN pairs to contiguous sequences flanking a target region causes dimerization of the FokI domain and ultimately a targeted DNA DSB, whereas CRISPR/Cas9 develops DSBs at a particular sequence [82]. The subsequent DSB is corrected using error-prone NHEJ or high-fidelity homology-directed repair (HDR) in the presence of a homologous DNA donor template [83].

To modify pathogenic mutations, an exogenous donor fragment is applied as a pattern. The major obstacle for developing an ideal HDR-based gene repair procedure is selection of the optimum exogenous template. The eligible donor fragment is transferred to HSPCs via various means including transfection as single-stranded oligodeoxynucleotide (ssODN), transduction using a viral vector-like integrase-defective lentiviruses (IDLVs) or recombinant adeno-associated viral vectors serotype 6 (rAAV6). The rAAV6 has long been considered as the most effective system for HDR-mediated gene repair transferring to HSPCs [84,85]. Despite rAAV6 efficiency in vitro, recent comparative studies revealed HSC engraftment decrease in animal models of immunodeficiency using rAAV6 compared to ssODNs [86,87].

HDR-based gene repair strategy could be efficiently applied in SCD as a monogenic and prevalent model. In the last decade, various strategies including ZFNs, TALENs, and CRISPR/Cas9 have been applied to induce HDR-mediated gene repair in order to correct the pathogenic variant in pluripotent stem cells (iPSCs) [88,89] and SCD patient-derived HSPCs (Figure 4) [90,91]. On the other hand, in vitro studies revealed that the correction efficiency was 7% to 50% (based on various editing technology and/or donor delivery systems), which is appropriate for HbA production up to 50% and improving the SCD phenotype [92]. However, transplantation of edited SCD cells presented a reduced gene repair frequency up to 10% when applied in vivo [93]. The incomplete HDR-mediated gene repair and the minority of HSCs in HSPC population are the main known reasons for insufficiency of HDR gene repair system in vivo. This led to generating an appropriate level of NHEJ-mediated INDELs in the *HBB* gene, which causes the *S-globin* gene to inactivate and leads to an appearance of undesired β-thalassemic phenotype [94].

HDR-mediated gene repair approaches have also been utilized to correct some mutations of β-thalassemia. Several studies have been performed on modifying specific point mutations of β-thalassemia in the patient derived iPSCs [95,96,97,98,99,100]. In a noticeable study, a wild-type *HBB* gene-complementary DNA is considered as a donor template for targeted integration. This plan is capable of repairing various point mutations involved in β-thalassemia [101]. However, the main limitation of this strategy is deficiency of a sufficient protocol for the production of adequate population enriched for HSCs as described earlier.

Regarding the inefficiency of HDR-based repair system, the non-specific NHEJ strategy was applied for long-term repopulation of HSCs. The imprecise disruption of DNA using NHEJ generated mutations in the non-coding region of the HBB gene, including IVS1-110G > A and IVS2-654C > T. These mutations were associated with the generation of new binding sites in the *HBB* introns, which led to synthesis of unusual mRNA and formation of immature termination codon [102].

In parallel studies conducted in 2019, TALEN- and CRISPR/Cas9-based editing strategies were applied for disruption of the unusual binding sites created by the mutations of IVS1-110G > A and IVS2-654C > T in patient HSPCs, which led to normal HBB binding and eventually elevated the expression of *HbA* gene [102,103].

Recently, a pair of engineered TALENs were used in order to induce targeted integration of a full-length β-globin complementary DNA subsequent to mutation induction of about 50% of human β-globin alleles near the site of the sickle cell-related mutation (Figure 4) [81].

In line with β-thalassemia studies, Ma et al. combined two methods of TALEN-based repair strategy of *HBB* mutations with effective production of edited patient-specific β-thalassemia iPSCs. Based on pluripotency, normal karyotype, lack of off-targets, and capability of iPSCs to differentiate into hematopoietic progenitor cells and further to erythroblasts with normal β-globin expression, this strategy seems the most ideal approach among GETs for further clinical applications [104].

In research published in 2021, the b039 thalassemia mutation was corrected using CRISPR-Cas9 technology. Consequently, erythroid progenitor cells formed and homozygous b039-thalassemia patients express normal *b-globin* genes. In terms of efficiency, a crucial point of this research is that the CRISPR Cas9-corrected cells have a significantly high production of HbA and were associated with a considerable reduction in free a-globin chains. The protocol could be the starting point for developing an efficient edition of CD34+ cells derived from b039 patients and designing combined therapies using the CRISPRCas9 editing of the *b-globin* gene [105].

## 6. Conclusions

Alongside the advances in gene therapy, gene editing tools offer an innovative approach for treating the β-hemoglobinopathies, which has recently emerged in clinical trials. Animal and human studies have presented the efficacy of the gene therapy approaches, currently based on GETs, in particular for β-thalassemia. Recent advances in genome sequencing methods, improvements in gene delivery systems, understanding the molecular mechanism involved in regulation of the *HBB* locus, and the progresses in gene-editing technology made a basis for new achievements in the treatment of hemoglobinopathies concomitant with significant clinical benefit.

Several significant challenges are yet to be effectively addressed for in vivo genome editing via CRISPR-Cas technology to be clinically translatable to different fields. CRISPR-Cas guide RNAs and nucleases must be optimized for potent and straightforward on-target consequences with minimal off-target consequences, and they should be delivered efficiently to specific human cells and have minimal antigenic properties so that they are accepted by human immune systems. Novel CRISPR-Cas enzymes and delivery systems are being developed to overcome these obstacles. To enhance the specificity of CRISPR-Cas9, researchers modified the Cas9 construct, optimized the configuration of sgRNA, and developed a CRISPR-Cas9 double nickase system that presents only single-strand nicks at target zones.

The more efficient approach using transient delivery systems instead of viruses for in vivo applications could reduce safety concerns and off-target effects of sustained expression of CRISPR editing components. Nanoparticles have been designed to deliver CRISPR components such as plasmid DNA, mRNA, and ribonucleoproteins, and polymeric formulations encapsulating chemotherapeutic agents could also be engineered to deliver CRISPR molecular components for combination therapy.

Hence, additional efforts are needed to address these problems, evaluate the safety of genome editing tools at the clinical level and follow the outcomes of GETs-mediated therapeutic approaches in related patients.

## Data Availability

Not applicable.

## Abbreviations

BCL11A	BAF Chromatin Remodeling Complex Subunit BCL11A
BDDs	blood disease disorders
BMT	bone marrow transplantation
Cas	CRISPR-associated protein
CRISPR	clustered regularly interspaced short palindromic repeat
crRNA	sequence-specific CRISPR RNA
EPO	erythropoietin
FOG	Friend of GATA
FOP	Friend of Prmt1
GATA-1	GATA-binding factor 1
GATA-2	GATA-binding factor 2
GVHD	graft versus host disease
GETs	gene editing tools
HbF	hemoglobin F
HDR	homology-directed repair
HLA	human leukocyte antigen
HPFH	hereditary persistence of fetal hemoglobin
HSCs	hematopoietic stem cells
IDLVs	integrase-defective lentiviruses
iPSCs	in pluripotent stem cells
KLF1	Kruppel Like Factor 1
NF-E2	Nuclear Factor, Erythroid 2
NHEJ	non-homologous end joining
rAAV6	recombinant adeno-associated viral vectors serotype 6
RBCs	red blood cells
SCD	sickle cell disease
SCL	Stem Cell Leukemia
SOX6	SRY-Box Transcription Factor 6
ssODN	single-stranded oligodeoxynucleotide
TALENs	transcription activator-like effector nucleases
tracrRNA	trans-activating crRNA
ZBTB7A	Zinc Finger and BTB Domain Containing 7A
ZFNs	zinc-finger nucleases

## References

[1] Mansilla-Soto J., Riviere I., Boulad F., Sadelain M. (2016). Cell and Gene Therapy for the Beta-Thalassemias: Advances and Prospects. Hum. Gene Ther..

[2] de Dreuzy E., Bhukhai K., Leboulch P., Payen E. (2016). Current and future alternative therapies for beta-thalassemia major. Biomed. J..

[3] Rachmilewitz E.A., Giardina P.J. (2011). How I treat thalassemia. Blood.

[4] Origa R., Adam M.P., Ardinger H.H., Pagon R.A., Wallace S.E., Bean L.J.H., Mirzaa G., Amemiya A. (1993–2022). Beta-Thalassemia. GeneReviews((R)).

[5] Taishikhina I., Lokhmatova M., Shelikhova L. (2020). Hematopoietic stem cell transplantation in patients with transfu-sion-dependent β-thalassemia. Review article. Pediatric Hematol. Oncol. Immunopathol..

[6] Khandros E., Kwiatkowski J.L. (2019). Beta thalassemia: monitoring and new treatment approaches. Hematol. Oncol. Clin..

[7] Thein S.L. (2018). Molecular basis of β thalassemia and potential therapeutic targets. Blood Cells Mol. Dis..

[8] Shah F.T., Sayani F., Trompeter S., Drasar E., Piga A. (2019). Challenges of blood transfusions in β-thalassemia. Blood Rev..

[9] Sadeghi M.M., Shariati L., Hejazi Z., Shahbazi M., Tabatabaiefar M.A., Khanahmad H. (2017). Inducing indel mutation in the *SOX6* gene by zinc finger nuclease for gamma reactivation: An approach towards gene therapy of beta thalassemia. J. Cell. Biochem..

[10] Ashrafizadeh M., Delfi M., Hashemi F., Zabolian A., Saleki H., Bagherian M., Azami N., Farahani M.V., Sharifzadeh S.O., Hamzehlou S. (2021). Biomedical application of chi-tosan-based nanoscale delivery systems: Potential usefulness in siRNA delivery for cancer therapy. Carbohydr. Polym..

[11] Kumar K.S., Girish Y.R., Ashrafizadeh M., Mirzaei S., Rakesh K.P., Gholami M.H., Zabolian A., Hushmandi K., Orive G., Kadumudi F.B. (2021). AIE-featured tetraphenylethylene nanoarchitectures in biomedical application: Bioimaging, drug delivery and disease treatment. Coord. Chem. Rev..

[12] Mirzaei S., Gholami M.H., Hashemi F., Zabolian A., Farahani M.V., Hushmandi K., Zarrabi A., Goldman A., Ashrafizadeh M., Orive G. (2022). Advances in understanding the role of P-gp in doxorubicin resistance: Molecular pathways, therapeutic strategies, and prospects. Drug Discov. Today.

[13] Keservani R., Sharma A.K. (2021). Nanoconjugate Nanocarriers for Drug Delivery.

[14] Liu D., Zhang H., Fontana F., Hirvonen J.T., Santos H.A. (2018). Current developments and applications of microfluidic technology toward clinical translation of nanomedicines. Adv. Drug Deliv. Rev..

[15] Zhang N., Wei M.-Y., Ma Q. (2019). Nanomedicines: A Potential Treatment for Blood Disorder Diseases. Front. Bioeng. Biotechnol..

[16] Uchida S., Perche F., Pichon C., Cabral H. (2020). Nanomedicine-Based Approaches for mRNA Delivery. Mol. Pharm..

[17] Patra J.K., Das G., Fraceto L.F., Campos E.V.R., del Pilar Rodriguez-Torres M., Acosta-Torres L.S., Diaz-Torres L.A., Grillo R., Swamy M.K., Sharma S. (2018). Nano based drug delivery systems: Recent developments and future prospects. J. Nanobiotechnol..

[18] Kunz J.B., Kulozik A.E. (2020). Gene Therapy of the Hemoglobinopathies. HemaSphere.

[19] Thein S.L. (2013). The molecular basis of β-thalassemia. Cold Spring Harb. Perspect. Med..

[20] Tari K., Valizadeh Ardalan P., Abbaszadehdibavar M., Atashi A., Jalili A., Gheidishahran M. (2018). Thalassemia an update: molecular basis, clinical features and treatment. Int. J. Biomed. Public Health.

[21] Mettananda S., Higgs D.R. (2018). Molecular Basis and Genetic Modifiers of Thalassemia. Hematol. Clin. N. Am..

[22] McGann P.T., Nero A.C., Ware R.E. (2017). Clinical features of β-thalassemia and sickle cell disease. Gene and Cell Therapies for Beta-Globinopathies.

[23] De Sanctis V., Kattamis C., Canatan D., Soliman A.T., Elsedfy H., Karimi M., Daar S., Wali Y., Yassin M., Soliman N. (2017). β-thalassemia distribution in the old world: an ancient disease seen from a historical standpoint. Mediterr. J. Hematol. Infect. Dis..

[24] Farashi S., Harteveld C.L. (2018). Molecular basis of α-thalassemia. Blood Cells Mol. Dis..

[25] Gupta S.K., Shukla P. (2016). Gene editing for cell engineering: trends and applications. Crit. Rev. Biotechnol..

[26] Kazemi B., Hosseini N., Khanahmad H., Esfahani B.N., Bandehpour M., Shariati L., Zahedi N. (2020). Targeting of cholera toxin A (ctxA) gene by zinc finger nuclease: pitfalls of using gene editing tools in prokaryotes. Res. Pharm. Sci..

[27] Carroll D. (2011). Genome engineering with zinc-finger nucleases. Genetics.

[28] Cai M., Yang Y. (2014). Targeted genome editing tools for disease modeling and gene therapy. Curr. Gene Ther..

[29] Ousterout D.G., Gersbach C.A. (2016). The Development of TALE Nucleases for Biotechnology. TALENs.

[30] Sun N., Zhao H. (2013). Transcription activator-like effector nucleases (TALENs): A highly efficient and versatile tool for genome editing. Biotechnol. Bioeng..

[31] Mohammadinejad R., Sassan H., Pardakhty A., Hashemabadi M., Ashrafizadeh M., Dehshahri A., Mandegary A. (2020). ZEB1 and ZEB2 gene editing mediated by CRISPR/Cas9 in A549 cell line. Bratisl. Med. J..

[32] Mohammadinejad R., Dehshahri A., Sassan H., Behnam B., Ashrafizadeh M., Gholami A.S., Pardakhty A., Mandegary A. (2020). Preparation of carbon dot as a potential CRISPR/Cas9 plasmid delivery system for lung cancer cells. Minerva Biotecnol..

[33] Barrangou R., Horvath P. (2017). A decade of discovery: CRISPR functions and applications. Nat. Microbiol..

[34] Knott G.J., Doudna J.A. (2018). CRISPR-Cas guides the future of genetic engineering. Science.

[35] Gupta R.M., Musunuru K. (2014). Expanding the genetic editing tool kit: ZFNs, TALENs, and CRISPR-Cas9. J. Clin. Investig..

[36] Ali G., Tariq M.A., Shahid K., Ahmad F., Akram J. (2020). Advances in genome editing: the technology of choice for precise and efficient β-thalassemia treatment. Gene Ther..

[37] Sripichai O., Fucharoen S. (2016). Fetal hemoglobin regulation in β-thalassemia: heterogeneity, modifiers and therapeutic ap-proaches. Expert Rev. Hematol..

[38] Musallam K.M., Sankaran V.G., Cappellini M.D., Duca L., Nathan D.G., Taher A.T. (2012). Fetal hemoglobin levels and morbidity in untransfused patients with β-thalassemia intermedia. Blood.

[39] Steinberg M.H. (2022). Targeting fetal hemoglobin expression to treat β hemoglobinopathies. Expert Opin. Ther. Targets.

[40] Demirci S., Leonard A., Tisdale J.F. (2020). Genome editing strategies for fetal hemoglobin induction in beta-hemoglobinopathies. Hum. Mol. Genet..

[41] Cui S., Engle J.D., Malik P.T.J. (2017). Reactivation of fetal hemoglobin for treating β-thalassemia and sickle cell disease. Gene and Cell Therapies for Beta-Globinopathies.

[42] Rivers A., Molokie R., Lavelle D. (2019). A new target for fetal hemoglobin reactivation. Haematologica.

[43] Topfer S.K., Feng R., Huang P., Ly L.C., Martyn G.E., Blobel G.A., Weiss M.J., Quinlan K.G.R., Crossley M. (2022). Dis-rupting the adult globin promoter alleviates promoter competition and reactivates fetal globin gene expression. Blood J. Am. Soc. Hematol..

[44] Ravi N.S., Wienert B., Wyman S.K., Bell H.W., George A., Mahalingam G., Vu J.T., Prasad K., Bandlamudi B.P., Devaraju N. (2022). Identification of novel HPFH-like mutations by CRISPR base editing that elevate the expression of fetal he-moglobin. Elife.

[45] Weber L., Frati G., Felix T., Hardouin G., Casini A., Wollenschlaeger C., Meneghini V., Masson C., De Cian A., Chalumeau A. (2020). Editing a γ-globin repressor binding site restores fetal hemoglobin synthesis and corrects the sickle cell disease phenotype. Sci. Adv..

[46] Cavazzana M., Mavilio F. (2018). Gene Therapy for Hemoglobinopathies. Hum. Gene Ther..

[47] Uda M., Galanello R., Sanna S., Lettre G., Sankaran V.G., Chen W., Usala G., Busonero F., Maschio A., Albai G. (2008). Genome-wide association study shows BCL11A associated with persistent fetal hemoglobin and amelioration of the phe-notype of β-thalassemia. Proc. Natl. Acad. Sci. USA.

[48] Xu J., Peng C., Sankaran V.G., Shao Z., Esrick E.B., Chong B.G., Ippolito G.C., Fujiwara Y., Ebert B.L., Tucker P.W. (2011). Correction of Sickle Cell Disease in Adult Mice by Interference with Fetal Hemoglobin Silencing. Science.

[49] Basak A., Hancarova M., Ulirsch J.C., Balci T.B., Trkova M., Pelisek M., Vlckova M., Muzikova K., Cermak J., Trka J. (2015). BCL11A deletions result in fetal hemoglobin persis-tence and neurodevelopmental alterations. J. Clin. Investig..

[50] Liu P., Keller J.R., Ortiz M., Tessarollo L., Rachel R.A., Nakamura T., Jenkins N.A., Copeland N.G. (2003). Bcl11a is essential for normal lymphoid development. Nat. Immunol..

[51] Sankaran V.G., Xu J., Ragoczy T., Ippolito G.C., Walkley C., Maika S.D., Fujiwara Y., Ito M., Groudine M., Bender M.A. (2009). Developmental and species-divergent globin switching are driven by BCL11A. Nature.

[52] Tsang J.C.H., Yu Y., Burke S., Buettner F., Wang C., Kolodziejczyk A.A., Teichmann S.A., Lu L., Liu P. (2015). Single-cell transcriptomic reconstruction reveals cell cycle and multi-lineage differentiation defects in Bcl11a-deficient hematopoietic stem cells. Genome Biol..

[53] Luc S., Huang J., McEldoon J.L., Somuncular E., Li D., Rhodes C., Mamoor S., Hou S., Xu J., Orkin S.H. (2016). Bcl11a De-ficiency Leads to Hematopoietic Stem Cell Defects with an Aging-like Phenotype. Cell Rep..

[54] Canver M.C., Smith E.C., Sher F., Pinello L., Sanjana N.E., Shalem O., Chen D.D., Schupp P.G., Vinjamur D.S., Garcia S.P. (2015). BCL11A enhancer dissection by Cas9-mediated in situ saturating mutagenesis. Nature.

[55] Vierstra J., Reik A., Chang K.-H., Stehling-Sun S., Zhou Y.-Y., Hinkley S.J., Paschon D.E., Zhang L., Psatha N., Bendana Y.R. (2015). Functional footprinting of regulatory DNA. Nat. Methods.

[56] Bauer D.E., Kamran S.C., Lessard S., Xu J., Fujiwara Y., Lin C., Shao Z., Canver M.C., Smith E.C., Pinello L. (2013). An Erythroid Enhancer of *BCL11A* Subject to Genetic Variation Determines Fetal Hemoglobin Level. Science.

[57] Chang K.-H., Smith S.E., Sullivan T., Chen K., Zhou Q., West J.A., Liu M., Liu Y., Vieira B.F., Sun C. (2017). Long-Term Engraftment and Fetal Globin Induction upon BCL11A Gene Editing in Bone-Marrow-Derived CD34 + Hematopoietic Stem and Progenitor Cells. Mol. Ther. Methods Clin. Dev..

[58] Khosravi M.A., Abbasalipour M., Concordet J.-P., Berg J.V., Zeinali S., Arashkia A., Azadmanesh K., Buch T., Karimipoor M. (2019). Targeted deletion of BCL11A gene by CRISPR-Cas9 system for fetal hemoglobin reactivation: A promising approach for gene therapy of beta thalassemia disease. Eur. J. Pharmacol..

[59] Psatha N., Reik A., Phelps S., Zhou Y., Dalas D., Yannaki E., Levasseur D.N., Urnov F.D., Holmes M.C., Papayannopoulou T. (2018). Disruption of the BCL11A Erythroid Enhancer Reactivates Fetal Hemoglobin in Erythroid Cells of Patients with β-Thalassemia Major. Mol. Ther. Methods Clin. Dev..

[60] Frangoul H., Altshuler D., Cappellini M.D., Chen Y.S., Domm J., Eustace B.K., Foell J., de la Fuente J., Grupp S., Handgretinger R. (2021). CRISPR-Cas9 Gene Editing for Sickle Cell Disease and beta-Thalassemia. N. Engl. J. Med..

[61] Ma S.-P., Gao X.-X., Zhou G.-Q., Zhang H.-K., Yang J.-M., Wang W.-J., Song X.-M., Chen H.-Y., Lu D.-R. (2022). Reactivation of γ-globin expression using a minicircle DNA system to treat β-thalassemia. Gene.

[62] Xu J., Sankaran V.G., Ni M., Menne T.F., Puram R.V., Kim W., Orkin S.H. (2010). Transcriptional silencing of γ-globin by BCL11A involves long-range interactions and cooperation with SOX6. Genes Dev..

[63] Yi Z., Cohen-Barak O., Hagiwara N., Kingsley P.D., Fuchs D.A., Erickson D.T., Epner E.M., Palis J., Brilliant M.H. (2006). Sox6 directly silences epsilon globin expression in definitive erythropoiesis. PLoS Genet.

[64] Shariati L., Rohani F., Heidari Hafshejani N., Kouhpayeh S., Boshtam M., Mirian M., Rahimmanesh I., Hejazi Z., Mo-darres M., Pieper I.L. (2018). Disruption of SOX6 gene using CRISPR/Cas9 technology for gamma-globin reactivation: An ap-proach towards gene therapy of β-thalassemia. J. Cell. Biochem..

[65] Maeda T., Ito K., Merghoub T., Poliseno L., Hobbs R.M., Wang G., Dong L., Maeda M., Dore L.C., Zelent A. (2009). LRF Is an Essential Downstream Target of GATA1 in Erythroid Development and Regulates BIM-Dependent Apoptosis. Dev. Cell.

[66] Lunardi A., Guarnerio J., Wang G., Maeda T., Pandolfi P.P. (2013). Role of LRF/Pokemon in lineage fate decisions. Blood.

[67] Masuda T., Wang X., Maeda M., Canver M.C., Sher F., Funnell A.P.W., Fisher C., Suciu M., Martyn G.E., Norton L.J. (2016). Transcription factors LRF and BCL11A independently repress expression of fetal hemoglobin. Science.

[68] Zhou D., Liu K., Sun C.-W., Pawlik K.M., Townes T.M. (2010). KLF1 regulates BCL11A expression and γ-to β-globin gene switching. Nat. Genet..

[69] Shariati L., Khanahmad H., Salehi M., Hejazi Z., Rahimmanesh I., Tabatabaiefar M.A., Modarressi M.H. (2016). Genetic dis-ruption of the KLF1 gene to overexpress the γ-globin gene using the CRISPR/Cas9 system. J. Gene Med..

[70] Lamsfus-Calle A., Daniel-Moreno A., Antony J.S., Epting T., Heumos L., Baskaran P., Admard J., Casadei N., Latifi N., Siegmund D.M. (2020). Comparative targeting analysis of KLF1, BCL11A, and HBG1/2 in CD34+ HSPCs by CRISPR/Cas9 for the induction of fetal hemoglobin. Sci. Rep..

[71] Siatecka M., Bieker J.J. (2011). The multifunctional role of EKLF/KLF1 during erythropoiesis. Blood.

[72] Arnaud L., Saison C., Helias V., Lucien N., Steschenko D., Giarratana M.-C., Prehu C., Foliguet B., Montout L., de Brevern A.G. (2010). A Dominant Mutation in the Gene Encoding the Erythroid Transcription Factor KLF1 Causes a Congenital Dyserythropoietic Anemia. Am. J. Hum. Genet..

[73] Wienert B., Martyn G.E., Funnell A.P.W., Quinlan K.G.R., Crossley M. (2018). Wake-up Sleepy Gene: Reactivating Fetal Globin for beta-Hemoglobinopathies. Trends Genet. TIG.

[74] Forget B.G. (1998). Molecular Basis of Hereditary Persistence of Fetal Hemoglobin. Ann. N. Y. Acad. Sci..

[75] Wienert B., Martyn G., Kurita R., Nakamura Y., Quinlan K.G.R., Crossley M. (2017). KLF1 drives the expression of fetal hemoglobin in British HPFH. Blood.

[76] Wienert B., Funnell A.P.W., Norton L., Pearson R.C.M., Wilkinson-White L.E., Lester K., Vadolas J., Porteus M.H., Matthews J., Quinlan K. (2015). Editing the genome to introduce a beneficial naturally occurring mutation associated with increased fetal globin. Nat. Commun..

[77] El-Beshlawy A., Mostafa A., Youssry I., Gabr H., Mansour I.M., El-Tablawy M., Aziz M., Hussein I.R. (2008). Correction of aberrant pre-mRNA splicing by antisense oligonucleotides in beta-thalassemia Egyptian patients with IVSI-110 mutation. J. Pediatr. Hematol. Oncol..

[78] Gabr H., El Ghamrawy M.K., Almaeen A.H., Abdelhafiz A.S., Hassan A.O.S., El Sissy M.H. (2020). CRISPR-mediated gene modification of hematopoietic stem cells with beta-thalassemia IVS-1-110 mutation. Stem Cell Res. Ther..

[79] Martyn G., Wienert B., Yang L., Shah M., Norton L.J., Burdach J., Kurita R., Nakamura Y., Pearson R.C.M., Funnell A.P.W. (2018). Natural regulatory mutations elevate the fetal globin gene via disruption of BCL11A or ZBTB7A binding. Nat. Genet..

[80] Giardine B., van Baal S., Kaimakis P., Riemer C., Miller W., Samara M., Kollia P., Anagnou N.P., Chui D.H., Wajcman H. (2007). HbVar database of human hemoglobin variants and thalassemia mutations: 2007 update. Hum. Mutat..

[81] Voit R.A., Hendel A., Pruett-Miller S.M., Porteus M.H. (2013). Nuclease-mediated gene editing by homologous recombination of the human globin locus. Nucleic Acids Res..

[82] Broeders M., Herrero-Hernandez P., Ernst M.P., van der Ploeg A.T., Pijnappel W.P. (2019). Sharpening the Molecular Scissors: Advances in Gene-Editing Technology. iScience.

[83] Tang X.-D., Gao F., Liu M.-J., Fan Q.-L., Chen D.-K., Ma W.-T. (2019). Methods for Enhancing Clustered Regularly Interspaced Short Palindromic Repeats/Cas9-Mediated Homology-Directed Repair Efficiency. Front. Genet..

[84] Schiroli G., Conti A., Ferrari S., DELLA Volpe L., Jacob A., Albano L., Beretta S., Calabria A., Vavassori V., Gasparini P. (2019). Precise Gene Editing Preserves Hematopoietic Stem Cell Function following Transient p53-Mediated DNA Damage Response. Cell Stem Cell.

[85] Wang J., Exline C.M., Declercq J.J., Llewellyn G.N., Hayward S.B., Li P.W.-L., Shivak D.A., Surosky R.T., Gregory P., Holmes M.C. (2015). Homology-driven genome editing in hematopoietic stem and progenitor cells using ZFN mRNA and AAV6 donors. Nat. Biotechnol..

[86] Pattabhi S., Lotti S.N., Berger M.P., Singh S., Lux C., Jacoby K., Lee C., Negre O., Scharenberg A.M., Rawlings D.J. (2019). In Vivo Outcome of Homology-Directed Repair at the HBB Gene in HSC Using Alternative Donor Template Delivery Methods. Mol. Ther. Nucleic Acids.

[87] Romero Z., Lomova A., Said S., Miggelbrink A., Kuo C.Y., Campo-Fernandez B., Hoban M.D., Masiuk K.E., Clark D.N., Long J. (2019). Editing the Sickle Cell Disease Mutation in Human Hematopoietic Stem Cells: Comparison of Endonucleases and Homologous Donor Templates. Mol. Ther..

[88] Martin R.M., Ikeda K., Cromer M.K., Uchida N., Nishimura T., Romano R., Tong A.J., Lemgart V.T., Camarena J., Pavel-Dinu M. (2019). Highly Efficient and Marker-free Genome Editing of Human Pluripotent Stem Cells by CRISPR-Cas9 RNP and AAV6 Donor-Mediated Homologous Recombination. Cell Stem Cell.

[89] Park S., Gianotti-Sommer A., Molina-Estevez F.J., Vanuytsel K., Skvir N., Leung A., Rozelle S.S., Shaikho E., Weir I., Jiang Z. (2017). A Comprehensive, Ethnically Diverse Library of Sickle Cell Disease-Specific Induced Pluripotent Stem Cells. Stem Cell Rep..

[90] Hoban M.D., Cost G.J., Mendel M.C., Romero Z., Kaufman M.L., Joglekar A.V., Ho M., Lumaquin D., Gray D., Lill G.R. (2015). Correction of the sickle cell disease mutation in human hematopoietic stem/progenitor cells. Blood.

[91] DeWitt M.A., Magis W., Bray N.L., Wang T., Berman J.R., Urbinati F., Heo S.-J., Mitros T., Muñoz D.P., Boffelli D. (2016). Selection-free genome editing of the sickle mutation in human adult hematopoietic stem/progenitor cells. Sci. Transl. Med..

[92] Vakulskas C.A., Dever D.P., Rettig G.R., Turk R., Jacobi A.M., Collingwood M.A., Bode N.M., McNeill M.S., Yan S., Camarena J. (2018). A high-fidelity Cas9 mutant delivered as a ribonucleoprotein complex enables efficient gene editing in human hematopoietic stem and progenitor cells. Nat. Med..

[93] Park S.H., Lee C., Dever D.P., Davis T.H., Camarena J., Srifa W., Zhang Y., Paikari A., Chang A.K., Porteus M.H. (2019). Highly efficient editing of the β-globin gene in patient-derived hematopoietic stem and progenitor cells to treat sickle cell disease. Nucleic Acids Res..

[94] Magis W., DeWitt M.A., Wyman S.K., Vu J.T., Heo S.-J., Shao S.J., Hennig F., Romero Z.G., Campo-Fernandez B., Said S. (2022). High-level correction of the sickle mutation is amplified in vivo during erythroid differentiation. iScience.

[95] Xie F., Ye L., Chang J.C., Beyer A.I., Wang J., Muench M.O., Kan Y.W. (2014). Seamless gene correction of β-thalassemia mutations in patient-specific iPSCs using CRISPR/Cas9 and *piggyBac*. Genome Res..

[96] Song B., Fan Y., He W., Zhu D., Niu X., Wang D., Ou Z., Luo M., Sun X. (2015). Improved hematopoietic differentiation effi-ciency of gene-corrected beta-thalassemia induced pluripotent stem cells by CRISPR/Cas9 system. Stem Cells Dev..

[97] Xu P., Tong Y., Liu X.-Z., Wang T.-T., Cheng L., Wang B.-Y., Lv X., Huang Y., Liu D.-P. (2015). Both TALENs and CRISPR/Cas9 directly target the HBB IVS2–654 (C > T) mutation in β-thalassemia-derived iPSCs. Sci. Rep..

[98] Niu X., He W., Song B., Ou Z., Fan D., Chen Y., Fan Y., Sun X. (2016). Combining Single Strand Oligodeoxynucleotides and CRISPR/Cas9 to Correct Gene Mutations in β-Thalassemia-induced Pluripotent Stem Cells. J. Biol. Chem..

[99] Liu Y., Yang Y., Kang X., Lin B., Yu Q., Song B., Gao G., Chen Y., Sun X., Li X. (2017). One-Step Biallelic and Scarless Correction of a beta-Thalassemia Mutation in Patient-Specific iPSCs without Drug Selection. Mol. Ther. Nucleic Acids..

[100] Wattanapanitch M., Damkham N., Potirat P., Trakarnsanga K., Janan M., Kheolamai P., Klincumhom N., Is-saragrisil S. (2018). One-step genetic correction of hemoglobin E/beta-thalassemia patient-derived iPSCs by the CRISPR/Cas9 sys-tem. Stem Cell Res. Ther..

[101] Cai L., Bai H., Mahairaki V., Gao Y., He C., Wen Y., Jin Y.-C., Wang Y., Pan R.L., Qasba A. (2017). A Universal Approach to Correct Various *HBB* Gene Mutations in Human Stem Cells for Gene Therapy of Beta-Thalassemia and Sickle Cell Disease. Stem Cells Transl. Med..

[102] Xu S., Luk K., Yao Q., Shen A.H., Zeng J., Wu Y., Luo H.-Y., Brendel C., Pinello L., Chui D.H.K. (2019). Editing aberrant splice sites efficiently restores β-globin expression in β-thalassemia. Blood.

[103] Patsali P., Turchiano G., Papasavva P., Romito M., Loucari C.C., Stephanou C., Christou S., Sitarou M., Mussolino C., Cornu T.I. (2019). Correction of IVS I-110(G>A) β-thalassemia by CRISPR/Cas-and TALEN-mediated disruption of aberrant regulatory elements in human hematopoietic stem and progenitor cells. Haematologica.

[104] Ma N., Liao B., Zhang H., Wang L., Shan Y., Xue Y., Huang K., Chen S., Zhou X., Chen Y. (2013). Tran-scription activator-like effector nuclease (TALEN)-mediated gene correction in integration-free β-thalassemia induced plu-ripotent stem cells. J. Biol. Chem..

[105] Cosenza L.C., Gasparello J., Romanini N., Zurlo M., Zuccato C., Gambari R., Finotti A. (2021). Efficient CRISPR-Cas9-based genome editing of β-globin gene on erythroid cells from homozygous β039-thalassemia patients. Mol. Ther. Methods Clin. Dev..

